# Predictors of the Onset of Type 1 Diabetes Obtained from Real-World Data Analysis in Cancer Patients Treated with Immune Checkpoint Inhibitors

**DOI:** 10.31557/APJCP.2020.21.6.1697

**Published:** 2020-06

**Authors:** Shinya Takada, Hashishita Hirokazu, Kayo Yamagishi, Sato Hideki, Endo Masayuki

**Affiliations:** 1 *Department of Pharmacy, National Hospital Organization Hokkaido Cancer Center, Japan. *; 2 *Faculty of Pharmaceutical Sciences, Hokkaido University of Science, Japan. *

**Keywords:** Immune checkpoint inhibitor, Japanese Adverse Drug Event Report database, type 1 diabetes

## Abstract

Medications that target programmed cell death protein-1 (PD-1) have proven effective. However, blockade of PD-1/Programmed death-ligand 1(PD-L1) causes immune-related adverse events (irAEs). Characteristics of this irAE include many symptom, low in frequency, and difficulty in prevention. The key to a successful ICI-related treatment lies in the management of irAEs resulting from immune checkpoint inhibitor (ICI) treatment. Although it is difficult to predict irAE, we tried to extract features of irAE expression from analysis of real-world database. This study used data extracted from the Japan Adverse Drug Event Report (JADER) database to assess risk factors associated with serious side effects of irAE, type 1 diabetes (T1DM). The analysis targets were nivolumab, atezolizumab, durvalumab, and pembrolizumab, and the study period was from July 2014 to June 2019. Analysis of Japanese population data confirmed that being women and having melanoma were risk factors for developing ICI-related T1DM. Analysis using this database in combination with information on ICI-related T1DM provides information and guidelines that will help in the safer treatment of ICI in the future.

## Introduction

Treatments that target programmed cell death protein-1 (PD-1) have significantly improved the clinical outcomes in patients with various malignancies (Barroso-Sousa et al., 2018; Godwin et al., 2017). Blocking of PD-1/PD-L1 signaling using monoclonal antibodies results in reactivation of T-cell-mediated anti-tumor immunity, resulting in a continuous anti-cancer response. Adverse-effects associated with PD-1/PD-L1 blockade have been characterized by a distinct range of toxic effects, termed immune-related adverse events (irAEs). The key to the successful treatment of malignancies lies in the management of ICI treatment-associated irAEs. It is known that, although it is low, the occurrence of irAE varies. However, irAE can occasionally lead toextremely severe complications. For example, the frequency of type 1 diabetes mellitus (T1DM) is known to be less than 1%, and its incidence is rare, thus the underlying pathological mechanisms and risk factors for T1DM are unknown (Barroso-Sousa et al., 2018; Godwin et al., 2017). Although there is no clear difference between general type 1 fulminant diabetes and irAE-induced T1DM with respect to the symptoms, the risk factors and patient background associated with the onset differ; however, the details are not clear. A meta-analysis has been performed to investigate the development of ICI-induced T1DM. According to a previous report, the median T1DMafter anti-PD1/PD-L1 treatment was 49 days, and 71% of the onset cases occurred within three months after the beginning of the treatment (Akturk et al., 2019). By using JADER, a voluntary reporting system in various backgrounds, and analyzing large-scale data using actual data, we believe that risk factors for adverse effects can be determined. Therefore, we examined the risk of developing T1DM in Japanese patients - being treated with ICI - using Japanese Adverse Drug Event Report (JADER) database.

## Materials and Methods

*Database information*

**Figure 1:**
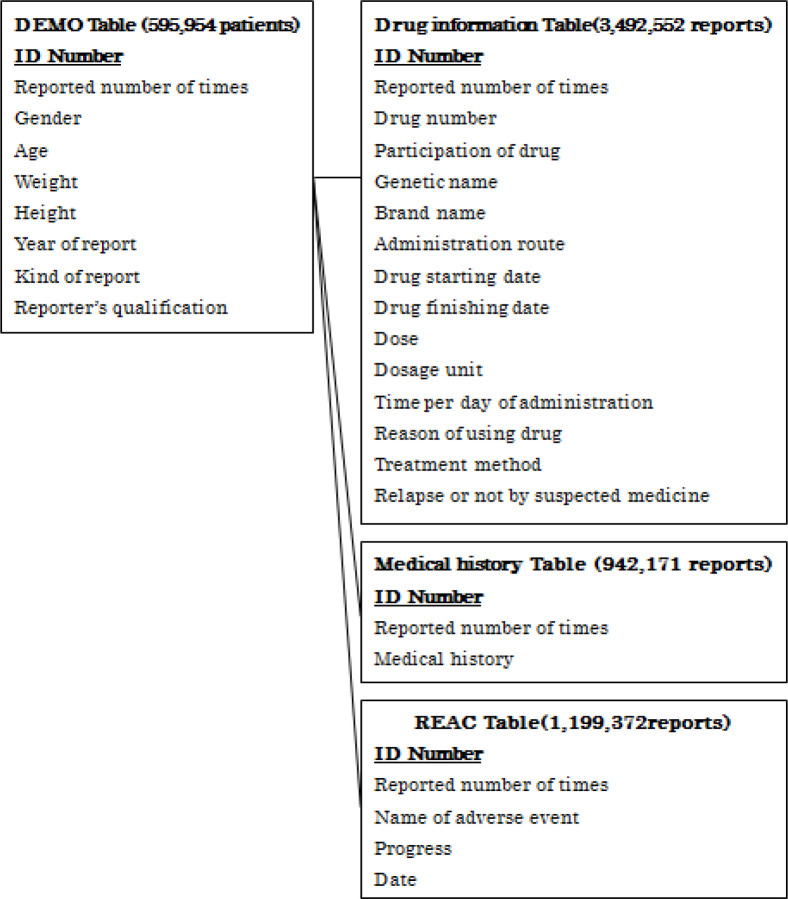
JADER Database Consisting of Four Information Tables (DEMO, DRUG, REAC, medical history).The bold number shows the number of reports extracted between July 2004 and June 2019. Abbreviations: DEMO, patient demographic information; DRUG, drug information; REAC, adverse events

Since April 2004, PMDA has reported cases of adverse events related to JADER drug therapy. This retrospective study employed JADER, an open-access database that serves as a voluntary report reporting system. The database consists of four data tables: patient demographics (demo), drug information (drugs), adverse events (reac), and primary illnesses (history) Figure 1. It renders informed consent unnecessary as information that can be used to identify individuals - such as patient name - is anonymized. The side effect report was downloaded from PMDA website (https://www.info.pmda.go.jp/fukusayoudb/CsvDownload.jsp), and the analyzed extraction period was from July 2014 to June 2019. We targeted four different ICI (nivolumab, atezolizumab, durvalumab, pembrolizumab). We extracted and analyzed the reports on the development of type 1 diabetes by ICI and non-ICI. And, a logistic regression analysis with age, sex, and cancer type as covariates of the risk of ICI-related T1DM were performed. This study was approved by the ethics committee of Hokkaido Cancer Center, Sapporo, Japan (Approval No: 31-57).

*Statistical analysis*

A chi-squared test analysis was used to compare the characteristics of patients. Multiple logistic regression analysis was used to extract risk factors for ICI-induced T1DM. Two-sided P-values less than 0.05 were considered significant. All analyses were performed using BellCurve for Excel (Social Survey Research Information Co., Ltd.).

**Table 1:** Type 1 Diabetes Mellitus in Patients Receiving Immune Checkpoint Inhibitor Therapy

ICIs	With ICIs	Without ICIs	Odds Ratio (95%CI)
	T1DM^#1^/Total cases	T1DM^#1^/Total cases	
Nivolmab	220/6301	706/580203	28.7(24.6-33.4)
Pembrolizumab	79/3499	706/586425	18.8(14.823.7)
Druvalumab	1/329	706/586175	2.5(0.4-14.3)
Atezolizumab	3/433	706/586071	5.8(1.9-17.0)
Total	303/10562	706/575942	23.4(20.4-26.8)

#1;T1DM, type 1 diabetes mellitus

**Table 2:** Multivariate Logistic Analysis of Patients with Type 1 Diabetes Mellitus

Characteristic	Total number of cases	Cases of T1DM	Odds Ratio(95%CI)
Sex (male=1,female=0)	2944	81	1.49(1.12-1.97)
Cancer Type (melanoma=1,other=0)	1251	47	2.01(1.44-2.82)
Age>70years (>70y=1,≤70=0)	5408	105	0.98(0.76-1.29)

## Results

As shown in Figure 1, after merging databases three tables using ID numbers (DRUG (3,492,552 reports), REAC (1,199,372 reports), and Medical history (942,171reports)), a total of 595,954 patients information were obtained. We extracted and analyzed the reports on the onset of type 1 diabetes by ICI and non-ICI.

*Comparison of ICI and non-ICI type 1 diabetes incidence*

The incidence of T1DM was significantly higher in patients with a history of ICI use, and the incidence in the ICI-free group was low (nivolumab: odds ratio 28.7 (24.6-33.4); pembrolizumab: odds ratio 18.8(14.823.7); atezolizumab: odds ratio 5.8(1.9-17.0); All ICI: odds ratio 23.4(20.4-26.8)).The use ofdurvalumabdid not differsignificantly from those of other ICIs (Table 1). 

*Multiple logistic regression analysis*

Multivariate logistic regression analysis showed that the risk of T1DMwas significantly higher in women (odds ratio, 1.49, 95% CI, 1.12-1.97, P <0.01) and melanoma (odds ratio, 2.01, 95% CI, 1.44-2.82, P <0.001) patients(Table 2).

## Discussion

Risk factors for the development of irAE - using clinical trial data - have not been identified. Our study analyzed real-world data obtained for the Japanese population and found that there is a specific risk of ICI-related T1DM. Several clinical trials using ICI apply strict inclusion criteria; however these criteria are later relaxed once the drug is made publicly available. Therefore, we believe that more accurate risk factors can be verified by analyzing real-world data. The data from the Japanese population confirmed an increased risk of developing ICI-related T1DM in women and melanoma. Whether sex differences are a risk factor for all irAEs remains unclear controversial. A study on sex differences confirmed that anti-programmed cell death protein 1 therapy is associated with high risk of endocrine disorders and pneumonia in women (Duma et al.2019). In addition, studies on cancer types have reported that women with metastatic melanoma and non-small cell lung cancer have a high incidence of irAEs (Wang et al., 2019).The human leukocyte antigen (HLA) genes are known to be related to T1DM. Among them, HLA-DQA1is a particularly important gene. Depending on a person’s genetic background, homozygosity at the HLA-DQA1 locus has been reported as a potential risk factor for developing melanoma (Planelles et al., 2006). Our study has several limitations. First, this study was a retrospective study that analyzed a database of prior information. Second, although some potential risk factors such HLA type (Atkinson et al., 2014), environment, and viral infection, are known to be associated with the development of T1DM, these could not be excluded in our study. Third, as the JADER database is based on self-reporting, so there is a possibility of reporting bias. Generally, mild side effects are rarely reported, and severe cases may be reported more frequently; such reporting bias is a feature of self-reporting databases. Moreover, though, information regarding age, sex, treatment history, complications and cancer type can be obtained from JADER, this information is limited. Furthermore, the JADER database consists of spontaneous reports and there is a lack of precise information for each patient, and inadequate reports may also be included, thus all confounders might not be completely excluded in our retrospective study. Therefore, a further detailed investigation is required. However, JADER simplifies risk assessment, and provides us with powerful information from real-world data. Using this method in combination with information regarding clinical practices related to ICI-related T1DM, this method can provide useful information for safe ICI treatment in the future. Therefore, a further detailed investigation is required. On the other hand, JADER considers it a useful method because it simplifies risk assessment and provides powerful information from actual data. 

In conclusion, we used a large JADER database to search for data on T1DM-related drugs and patient characteristics. As a result, women, and melanoma were identified as risk factors that induced ICI-related T1DM. Analyzing this database in combination with information on ICI-related T1DM may provide information and guidelines that may help in the safer treatment of ICI in the future.
